# Executive Functions, Psychiatric Symptoms and ADHD in Child Psychiatric Patients–Concurrent and Longitudinal Associations from Preschool to School Age

**DOI:** 10.1007/s10578-023-01635-5

**Published:** 2023-12-12

**Authors:** Tiina Seikku, Taru Saarelainen, Tiia Kuha, Katri Maasalo, Hanna Huhdanpää, Eeva T. Aronen

**Affiliations:** 1 Child Psychiatry, University of Helsinki and Helsinki University Hospital, Helsinki, Finland; 2 Pediatric Research Center, New Children’s Hospital, Helsinki University Hospital, Helsinki, Finland

**Keywords:** Executive functions, Psychiatric symptoms, Child psychiatry, ADHD

## Abstract

We investigated in a child psychiatric sample whether preschool age executive functions (EFs) associate with concurrent and school age psychiatric symptoms and ADHD diagnosis. At baseline the children (n = 172) were 4–7 years old, at follow-up (n = 65) 8–13 years. EFs were measured at baseline with Attention and Executive Function Rating Inventory—Preschool Version, psychiatric symptoms were measured at both timepoints by Child Behavior Checklist. Information on diagnoses was collected from medical records. Deficits in EFs were associated with more concurrent externalizing and attention symptoms, but less internalizing symptoms. Preschool EFs predicted only school age attention symptoms. Preschool EFs were associated with both concurrent and school age ADHD diagnosis. Our results emphasize the importance of recognizing EF deficits early to arrange appropriate support to reduce later problems. More research is needed to understand the role of EFs over time in the manifestation of psychiatric symptoms in child psychiatric patients.

## Introduction

Executive functions (EFs) are cognitive processes that begin to develop in early childhood and have an impact on a broad range of human functioning, including psychopathology. EFs encompass abilities such as inhibition, attentional regulation, working memory, and planning and monitoring of one’s behavior [1–4]. EFs are needed to adapt to the environment and to function flexibly. With deficits of EFs the adaptation becomes dysfunctional and inflexible, and problem behaviors develop instead [5]. Preschool age and the transition to formal education is a crucial period in the development of EFs, as not only the cognitive abilities of children take a leap [1, 2, 4, 6] but also the demands of the environment increase.

Deficits of EFs in preschool and school age children have been associated with a wide range of both externalizing [7, 8] and internalizing [9, 10] problems, as well as attention deficit hyperactivity disorder (ADHD) [7, 8]. When looking at separate components of EFs, deficits of inhibition appear to be consistently associated with increased externalizing problems [11–15] and ADHD [7, 16–19]. There is also evidence for the relationship between attentional deficits and externalizing problems [11] and ADHD [20, 21]. In addition, ADHD has been found to be associated with deficits in execution of action (including planning, initiating, and monitoring) and cognitive flexibility [16, 17]. Regarding internalizing problems, the results on individual components of EFs are mixed, although inhibition [9], cognitive flexibility [22–24] and attention [9, 11] may be relevant.

Previous research supports the predictive role of early EF deficits to later development of externalizing [10, 25, 26] and internalizing problems [8, 10, 24, 25, 27]. The research on the predictive ability of EFs on later ADHD is mixed. Generally, early deficits of EFs seem to be able to predict later ADHD [28–31], but when considering individual confounders, the association is weak [31] or EFs only predict inattentive ADHD symptoms [28]. Some studies have also been unable to find a longitudinal effect [32], or the direction of the effect is unclear [33].

Most studies have used community samples to examine the relationship between EFs and psychiatric symptoms (e.g., [11, 13, 14, 22–24]. Several studies (e.g., [13, 14, 34] have compared groups of children with clinical levels of symptoms to groups of children with subclinical symptoms, but the samples are still community-based. When community-based clinical and non-clinical symptom groups have been compared in meta-analyses, it appears that the association between EFs and externalizing symptoms is stronger in the children with clinical level symptoms [7, 8]. Earlier we have reported [35] that preschool age child psychiatric patients have consistently more EF deficits than children in a community sample. Besides our study [35], there are hardly any studies exclusively focusing on how the relationship between EFs and psychiatric symptoms manifests in child psychiatric patients, and even fewer that consider the full range of psychiatric symptoms, not just selected diagnostic groups. In addition, most studies focus on school aged children, even though preschool period is extremely relevant in the development of EFs.

Child psychiatric patients have an increased risk for future psychopathology [36–38], and therefore attempts to recognize the developmental paths preceding psychiatric symptoms are crucial for effective early interventions. Focusing on child psychiatric patients in general instead of diagnostic groups is beneficial for clinical work with young children, as disorders and symptoms tend to be comorbid [39] and many diagnoses are not received until later childhood. In addition, most psychiatric distress is dimensional in nature, especially in childhood, and the line between symptom types, as well as normal and abnormal, is vague [40]. Examining early developmental paths in child psychiatric patients can also reveal information on heterotypic continuation of psychopathology and the role of underlying core deficits, such as EFs, in it.

This follow-up study aims to fill the gap left by previous research, by investigating whether preschool age deficits of EFs are associated with concurrent and school age psychiatric symptoms and ADHD diagnosis in a child psychiatric sample. Based on previous research we hypothesized that there are both concurrent [7, 8, 10, 11] and longitudinal [8, 10, 24–27] associations between EFs and psychiatric symptoms, and specifically that deficits in inhibition and attention are associated with externalizing [11, 13–15] and internalizing problems [9, 11, 24, 41]. Regarding the association between EFs and ADHD, we hypothesized that total deficits in EF as well as in inhibition, attention and execution of action are associated with concurrent ADHD [7, 8, 16–21], while total EF predicts later ADHD diagnosis [28–31].

## Methods

### Participants

The baseline sample (n = 176, after excluding cases with missing values n = 172) included children from child psychiatric outpatient clinics at Helsinki University Hospital (Helsinki and Vantaa) between 2015 and 2017. The inclusion criteria were: (1) 4 to 7 years old, (2) Finnish-speaking parents, and (3) in daycare, as EFs at preschool age were reported by daycare teachers [42].

The follow-up sample was collected in 2021 by contacting families of the baseline sample via mail. Addresses were collected from The Digital and Population Data Services Agency in Finland and were available for 171 participants. Families received contact letters and questionnaires, and they were reminded up to three times if they did not respond. Five families declined to participate. A total of 69 families responded (response rate 40.4%) of which one case was excluded due to incomplete questionnaire with over half of the answers missing. Families were also requested to deliver an invitation to participate in the follow-up phase to the teachers of the children. Teachers’ part of the study included ATTEX questionnaire for school age children [21], but only 17 teachers responded, so the data were not used.

The participants in the follow-up sample (n = 68, after excluding cases with missing values n = 65) did not differ from the rest of the baseline sample (n = 107) in their demographic characteristics (age, gender, parents’ educational level, living situation; p > .10), sleep problems (p = .24), psychiatric symptoms (CBCL Total; p = .30), executive functioning (ATTEX-P total; p = .70) or ADHD diagnosis (p = .43) at baseline.

This study was granted ethical approval by the Helsinki University Hospital Ethics Committee. Written informed consent was obtained from the children’s caregivers.

### Measures

#### Background Information

Background information was collected both at baseline (T1) and follow-up (T2). Caregiver questionnaires included questions about children’s age, gender, and family structure (only T2), and parents’ educational level. Daycare teachers provided information on children’s special support in daycare (T1). Information on family structure at T1 was collected from medical records.

#### Information on Diagnoses and Medication

Information on children’s most recent diagnoses (ICD-10), both T1 and T2, were collected from the hospital medical records of the patients, as well as information on children’s medication at T1. At T2, for 13 children diagnoses were not available as their contact with hospital had finished. Children’s current medication at T2 was reported by the parents. Patients’ diagnoses were made by a child psychiatrist or a trainee in child psychiatry (supervised by a specialist) using ICD-10 classification of mental and behavioral disorders (World Health Organization, 2016). For the diagnostic decisions a comprehensive child psychiatric evaluation is made including anamnesis from the parents, child’s individual assessment, information from school or daycare (with permission from parents). The ADHD Rating Scale (ADHD-RS) is routinely used to get information on ADHD symptom severity. Other structured instruments, tests, examinations, consultations are used tailored individually by clinician in charge of the patient. For ADHD diagnosis National Guidelines for ADHD are followed (www.kaypahoito.fi/ADHD).

#### Executive Functions (EFs)

EFs at T1 were assessed by the Attention and Executive Function Rating Inventory – Preschool version (ATTEX-P) [43], filled in by daycare teachers. The questionnaire contains 44 questions answered with a 3-point Likert scale (0, not a problem; 1, sometimes a problem; 2, often a problem) and it is designed to be used for children aged 4–7 years in a daycare environment. The questions combine into a total score, with higher scores representing more EF problems, and nine clinical scales (distractibility, impulsivity, motor hyperactivity, directing attention, sustaining attention, shifting attention, initiative, planning and execution of action). The nine clinical scales represent three domains of EF: inhibition, attentional control, and execution of action ATTEX-P has shown good psychometric properties [43]. The internal consistency of ATTEX-P in the present study was excellent (Cronbach’s alpha 0.96; for inhibition, attentional control, and execution of action Cronbach’s alpha values were 0.95, 0.91, 0.83, respectively).

#### Psychiatric Symptoms

Caregivers filled in the standardized ASEBA Child Behavior Checklist (CBCL) form, for preschool children (CBCL/1½-5; [44] at T1, and school-aged children (CBCL/6–18; [45] at T2. The questionnaires comprise of 100 and 113 items, respectively, scored on a 3-point Likert scale (0, not true; 1, somewhat or sometimes true; 2, very true or often true). Higher scores reflect more problems. Both CBCL versions include Externalizing (in the present study Cronbach’s alpha 0.88) and Internalizing problems (Cronbach’s alpha 0.79) broadband scales, and a total problems score (Cronbach’s alpha 0.92). In addition, both CBCL versions include several narrowband symptom scales, of which Attention problems scale (Cronbach’s alpha 0.78) was used in this study. CBCL attentional problems scale comprises both inattentive and hyperactive/impulsive (ADHD) symptoms. In the preschool version the Attention scale together with Aggressive behavior scale comprise the Externalizing scale. In the school-age version the Externalizing scale is formed from Aggressive behavior and Rule-breaking behavior scales, and the Attention scale is not a part of it. Achenbach and Rescorla [44, 45]. Most scales of the preschool questionnaire are fairly comparable to scales of the school-age questionnaire, though the items in the scales are slightly different attuned to reflect age-appropriate behavior. The sum scores that have counterparts in both preschool and school-age versions are comparable and can be used in longitudinal research [44, 45]. The CBCL preschool and school-age scales used in the present study correlated statistically significantly (Total problems, *r =* .565, *p* < .001; Externalizing, *r =* .531, *p* < .001; Internalizing, *r =* .497, *p* < .001; Attentional problems, *r =* .247, *p* < .05).

### Data Analysis

#### Variables

EFs were analyzed as continuous variables on the level of the ATTEX-P total score and three subscales: inhibition (consisting of distractibility, impulsivity, and motor hyperactivity), attentional control (consisting of directing attention, sustaining attention, and shifting attention), and execution of action (consisting of initiative, planning, and execution of action). The total score is the sum of the three subscale scores.

Psychiatric symptoms were analyzed as continuous variables on the level of the CBCL total score, the broadband problem scales (internalizing and externalizing) and one narrowband symptom scale (attention symptoms). The CBCL was scored using the ASEBA-web system, and raw scores were used in analyses, to best capture the full range of psychological symptoms.

Some of the variables were not normally distributed (at T1 internalizing symptoms, at T2 internalizing, externalizing and total symptoms), so a square root transformation was applied for these variables to reach normal distribution.

ADHD was analyzed as a dichotomous variable of verified ADHD diagnosis or no ADHD diagnosis (including at T2 children with no information on diagnoses).

#### Covariates

Variables with theoretical relevance or observed associations in prior research were selected for covariates. Background information regarding parents’ education (T1), child’s age (T1 and T2) and gender, and child’s sleep problems (T1) were used as covariates in the fully adjusted analyses. Parental education variable was formed by considering the highest education accomplished by either parent and coding these into high (lower higher education degree, or further) or low (upper secondary school, vocational college, or less) education. Sleep problems were assessed with the Sleep Disturbance Scale for Children (SDSC) [46], filled in by parents at T1. The scores of two subscales of SDSC were used in this study as covariates: (1) disorders of initiating and maintaining sleep (DIMS) and (2) sleep–wake transition disorders (SWTD). These sleep subscales were selected based on previous research linking them to attentional, externalizing, and internalizing problems [42].

#### Statistical Analyses

Statistical analyses were performed using IBM SPSS Statistics (Version 25). For drawing graphs, R Statistical Software (v.4.2.2; R Core Team [47]) and RStudio (v.12.0.353; Posit team [48]) were used.

At first, correlations between the variables (ATTEX-P scores, CBCL scores, ADHD diagnoses, control variables) were examined. To investigate concurrent and longitudinal associations between EFs and psychiatric symptoms, 32 linear regression models were built, where ATTEX-P total score or one of the three subscales predicted T1 or T2 CBCL total, externalizing, internalizing or attention score. In linear regression analyses the ATTEX-P raw scores were used. To investigate concurrent and longitudinal associations between EFs and ADHD, 8 logistic regression models were built, where ATTEX-P total score or one of the subscales predicted T1 or T2 ADHD diagnosis. In logistic regression analyses the standardized (M = 0, SD = 1) ATTEX-P scores were used to enable comparison of odds ratios (OR). Analyses investigating concurrent associations were conducted with the baseline sample (n = 172). Analyses investigating longitudinal associations were conducted with the follow-up sample (n = 65). All the analyses were performed both unadjusted and fully adjusted.

## Results

### Descriptive Data

At baseline (T1) 70,9% (n = 122) of the children were boys, age range was between 4 and 7 years (M = 5.7, SD = 0.7). At follow-up (T2) 73.8% (n = 48) of the children were boys, age range was 8–13 years (M = 11.0, SD = 1.1). Background information on parents’ educational level, family structure, child’s support in daycare or at school, medication, and ADHD diagnosis at T1 and T2, is presented in Table 1. Means, standard deviations, and range of ATTEX-P raw scores at T1 and CBCL raw and T-scores at T1 and T2 are presented in Table 2.

**Table 1:** Demographic information of families, daycare/school special support, medication, and ADHD diagnosis, at T1 (n = 172) and T2 (n = 65)

	Time 1, n (%)	Time 2, n (%)
Parents educational level		
High	86 (50.0)	37 (56.9)
Low	86 (50.0)	25 (38.5)^a^
Family structure		
Nuclear family	70 (40.7)	26 (40.0)
Living with one biological parent^b^	91 (52.9)	32 (49.2)
Other	11 (6.4)	7 (10.8)
Special support in daycare/special class in school	130 (75.6)	25 (38.5)
ADHD diagnosis (F90)	41 (23.8)	31 (47.7)
ADHD Medication	5 (2.9)	26 (40.0)

^a^In three cases the information on parents' educational level was missing. ^b^Includes children living in a blended family.

**Table 2:** Means, standard deviations, and ranges of ATTEX-P raw scores at T1 and CBCL raw and T-scores a at T1 (n = 172) and T2 (n = 65)

	T1			T1				T2			
	Raw scores			Raw scores		T-scores^a^		Raw scores		T-scores^a^	
ATTEX-P	M (SD)	Range	CBCL	M (SD)	Range	M (SD)	Range	M (SD)	Range	M (SD)	Range
Total score	41.0 (21.5)	0–82	Total score	62.4 (28.5)	5-138	63.0 (11.9)	32–90	52.7 (32.8)	3-139	62.1 (12.2)	34–84
Inhibition	20.4 (11.7)	0–40	Externalizing	23.5 (10.1)	2–46	62.5 (12.0)	35–95	14.7 (11.5)	0–49	59.3 (12.4)	33–84
Attention	11.4 (6.6)	0–26	Internalizing	18.8 (11.5)	0–56	62.0 (12.1)	29–90	14.1 (9.9)	0–43	62.1 (11.6)	34–89
Execution of action	9.1 (5.3)	0–22	Attention	4.4 (2.1)	0–10	59.9 (7.6)	50–80	8.3 (4.9)	0–17	63.6 (10.3)	50–90

^a^T-scores are standardized scores based on ASEBAs multicultural population norms, with cut-off points for subclinical (60–62) and clinical (63 or more) scores for total, externalizing and internalizing scales [44, 45].

### Preschool Age EFs and Concurrent and School Age Psychiatric Symptoms

Higher scores on ATTEX-P total, inhibition and execution of action were associated with higher CBCL externalizing scores (p = .01, p = .00, p = .03 respectively) at T1 (see Table 3). On the other hand, higher ATTEX-P total and inhibition scores were associated with lower internalizing scores (p = .05, p = .02, respectively) at T1. The associations remained significant after controlling for sleep problems, age, sex, and parental education (p < .05), except for the associations between ATTEX-P total and internalizing scores and between execution of action and externalizing scores (p = .07, p = .09, respectively) (see Table 4). Adding the covariates strengthened the association between ATTEX-P attention score and externalizing score (p = .07).

ATTEX-P total score and all the subscales (inhibition, attention, execution of action) were positively associated with CBCL attention scores at T1 (p < .001). The associations remained statistically significant after controlling for sleep problems, age, sex, and parental education (p < .01).

ATTEX-P total score or none of the subscales were associated with CBCL total score at T1.

ATTEX-P total score or none of the subscales predicted CBCL total or broadband scales at T2 (p > .05 for all). However, higher total, inhibition and execution of action scores predicted higher CBCL attention scores at T2, but only in the fully adjusted models (p = .04, p = .06, p = .05, respectively).

**Table 3:** Unadjusted regression models in investigating associations between ATTEX-P scores and CBCL scores at T1

	CBCL															
	Attention				Internalizing				Externalizing				Total			
ATTEX-P	B	95% CI	*p*	R^2^ *	B	95% CI	*p*	R^2^	B	95% CI	*p*	R^2^	B	95% CI	*p*	R^2^
Inhibition	0.06	0.04–0.09	< 0.001	0.12	− 0.02	− 0.04– − 0.00	0.02	0.03	0.20	0.07–0.33	0.00	0.05	− 0.01	− 0.38–0.36	0.95	− 0.01
Attention	0.09	0.04–0.13	< 0.001	0.07	− 0.02	− 0.06–0.01	0.12	0.01	0.19	− 0.04–0.42	0.10	0.01	− 0.12	− 0.77–0.54	0.73	− 0.01
Execution of action	0.10	0.05–0.16	< 0.001	0.06	− 0.02	− 0.06–0.02	0.42	− 0.00	0.32	0.04–0.61	0.03	0.02	0.25	− 0.57–1.06	0.55	− 0.00
Total score	0.03	0.02–0.05	< 0.001	0.11	− 0.01	− 0.02–0.00	0.05	0.02	0.10	0.03–0.17	0.01	0.04	0.00	− 0.20–0.20	0.99	− 0.01

* Adjusted R^2^ values

**Table 4:** Fully adjusted regression models with sleep, age, sex, and parental education controlled in investigating associations between ATTEX-P scores and CBCL scores at T1

	CBCL															
	Attention				Internalizing				Externalizing				Total			
ATTEX-P	B	95% CI	*p*	R^2^ *	B	95% CI	*p*	R^2^	B	95% CI	*p*	R^2^	B	95% CI	*p*	R^2^
Inhibition	0.05	0.03–0.08	< 0.001	0.20	− 0.02	− 0.04– − 0.01	0.01	0.26	0.15	0.03–0.28	0.02	0.28	− 0.07	− 0.40–0.27	0.69	0.37
Attention	0.08	0.04–0.13	< 0.001	0.20	− 0.01	− 0.04–0.02	0.50	0.22	0.20	− 0.02–0.41	0.07	0.27	0.20	− 0.36–0.75	0.49	0.37
Execution of action	0.08	0.02–0.14	0.01	0.18	− 0.01	− 0.05–0.03	0.54	0.22	0.23	− 0.03–0.49	0.09	0.27	0.25	− 0.57–1.06	0.46	0.37
Total score	0.03	0.01–0.04	< 0.001	0.21	− 0.01	− 0.02–0.00	0.07	0.24	0.08	0.01–0.15	0.02	0.28	0.02	− 0.16–0.19	0.85	0.37

* Adjusted R^2^ values

### Preschool Age EFs and Concurrent and School Age ADHD

Higher scores on ATTEX-P total and all the subscales (inhibition, attention, execution of action) were associated with increased probability of T1 ADHD diagnosis. The effects of inhibition (OR = 2.43, p < .001) and total score (OR = 2.34, p = .00) were larger than those of attention (OR = 1.59, p = .01) and execution of action (OR = 1.49, p = .03). After controlling for sleep problems, age, sex, and parental education the associations remained significant (p < .05), apart from execution of action (p = .13). (See Table 5.)

**Table 5:** Logistic regression models in investigating associations between ATTEX-P scores and ADHD diagnosis at T1 (n = 172) and T2 (n = 65).

	Unadjusted models				Fully adjusted models			
ATTEX-P	EXP(B)	95% CI	*p*	adj. R^2^	EXP(B)	95% CI	*p*	adj. R^2^
ADHD diagnosis (T1)								
Inhibition	2.43	1.58–3.73	< 0.001	0.16	2.02	1.24–3.29	0.00	0.27
Attention	1.59	1.10–2.31	0.01	0.05	1.59	1.00–2.51	0.05	0.24
Execution of action	1.49	1.03–2.14	0.03	0.04	1.39	0.91–2.13	0.13	0.22
Total score	2.34	1.31–4.18	0.00	0.12	1.89	1.16–3.06	0.01	0.26
ADHD diagnosis (T2)								
Inhibition	2.14	1.23–3.71	0.01	0.16	2.08	1.04–4.16	0.04	0.26
Attention	2.02	1.16–3.49	0.01	0.14	1.95	1.04–3.67	0.04	0.26
Execution of action	2.15	1.23–3.77	0.01	0.16	2.01	1.08–3.73	0.03	0.20
Total score	2.07	1.21–3.57	0.01	0.19	2.32	1.15–4.67	0.02	0.28

**Fig. 1:**
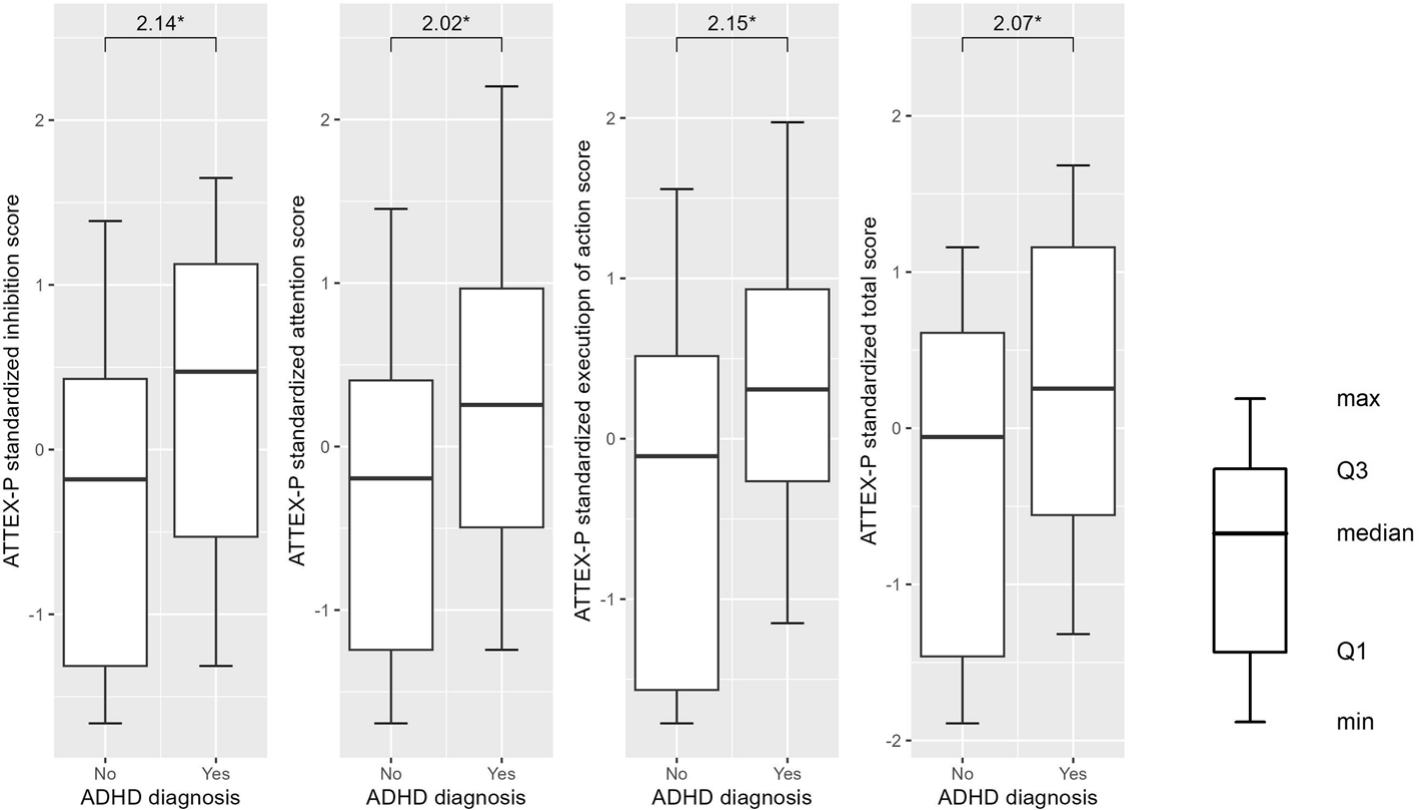
ATTEX-P scores at T1 for children with and without ADHD diagnosis at T2, *p < .05

Higher scores on ATTEX-P total and all the subscales also predicted increased probability of ADHD diagnosis at T2 (ORs between 2.02 and 2.15, p = .01). After controlling for sleep problems, age, sex, and parental education the associations remained significant (p < .05). (See Table 5.) Since there was a correlation between T1 and T2 ADHD diagnoses (r = .27, p = .03), an additional logistic regression analysis was performed for the association between ATTEX-P scores and T2 ADHD diagnosis, controlling for T1 diagnosis. The associations remained significant for ATTEX-P total score (OR = 1.83, p = .04) and execution of action (OR = 1.87, p = .03), but not for inhibition (OR = 1.67, p = .09) and attention (OR = 1.65, p = .07). Figure 1 shows ATTEX-P scores at preschool age in children with and without ADHD diagnosis at school age.

## Discussion

This study aimed to answer how preschool age EFs (including inhibition, attention, and execution of action) are associated with concurrent and school-age psychiatric symptoms (including internalizing, externalizing and attention symptoms) and ADHD diagnosis in child psychiatric patients.

In line with our hypothesis, deficits in preschool age total EFs and inhibition were associated with more externalizing symptoms concurrently. Similar results have been obtained in previous research [7, 8, 11, 13, 14, 49], although many of the studies reporting an association between inhibition and externalizing symptoms, have included ADHD symptomology in externalizing symptoms (e.g., [13, 14, 49]. As the preschool version of CBCL [44] includes attention symptoms in the externalizing scale, and the sample used in this study also included 23,8% children with ADHD diagnosis, the role of ADHD on externalizing symptoms cannot be ignored.

Deficits in total EF and inhibition were also associated with preschool age internalizing symptoms, but the effect was opposite from what we hypothesized: higher inhibition and executive functioning were associated with more internalizing symptoms. This is surprising, as previous literature has associated deficits of EF and inhibition with more internalizing symptoms [9, 24, 41]. Disparate results have been obtained, however. Eisenberg et al. [11] did not find children with internalizing symptoms to have lower inhibitory control, but instead lower impulsivity, than control children. The pattern of lower impulsivity/higher inhibition in internalizing children might describe a subset of child psychiatric patients with predominantly behaviorally inhibited temperament (see [50] or sluggish cognitive tempo (see [51]. It is possible, that in a clinical sample, deviations from optimal inhibition can be observed at both ends of continuum. Although [11] used a community sample, it was a selective sample with children having CBCL scores indicating at least a risk for problem behavior.

Execution of action as such has not been mentioned in previous studies, and studies using similar components of executive functions (planning, initiation, monitoring) have not analyzed these components separately from total EF (see [25, 26]. In this study, deficits in execution of action were associated with increased externalizing symptoms in preschool, although the association diminished after controlling for sleep, age, sex, and parental education. Execution of action is an essential part of everyday EF and should be included in future research to further examine its role in externalizing symptoms.

Attentional EF deficits have previously been associated with both internalizing and externalizing symptoms [9, 11]. In the present study, no association between attentional deficits and internalizing, externalizing or total symptoms at preschool age was found. It may be that combining directing, shifting, and sustaining attention into one category led to diverging results, as previous research has separately investigated attentional shifting [9, 11], sustained and selective attention [9]. It is also possible that recognizing attentional deficits in preschool age children is more difficult than with older children, and that impulsive and hyperactive behaviors may hide co-occurring attentional deficits. The deficits discernible with neuropsychological tests may not be as easily observed in everyday life.

This study utilized the CBCL attention symptom scale (includes both attention and hyperactivity problems) in addition to broadband scales (internalizing and externalizing). Deficits in total EFs and all the subscales were associated with increased attentional symptoms in preschool age, and the association appeared more straightforward than those with other psychiatric symptoms. In addition, CBCL attentional symptoms were the only school age symptom category that preschool executive functions were able to predict.

Attentional symptoms are a central part of ADHD symptomology, and they often have been studied as such, but usually not by themselves. One study [26] separately examined the associations of EFs and different externalizing symptoms, and found EF to predict later inattentive symptoms, but only in boys. In the present study boys and girls were not compared, but the large proportion of boys in the sample may reflect similar results. As with the association between inhibition and externalizing symptoms, the large proportion of children with ADHD in this sample may also explain the stronger association with attention symptoms.

It is noteworthy, that in the preschool version of CBCL [44], attention symptoms are part of externalizing problems broadband scale, and therefore the results are partly overlapping. The fact that CBCL attention symptoms had a stronger association with all the EFs than CBCL externalizing symptoms did, suggests that the effect seen on externalizing symptoms is mostly based on the association between EFs and CBCL attention symptoms. Similar pattern has been noted in prior research. For example, [52] and [28] have reported the association between EFs and oppositional defiant disorder/conduct disorder (ODD/CD) to disappear after controlling for ADHD symptoms. In a study [49] with a clinical sample of children with ADHD and/or disruptive behavior disorder (DBD) inhibitory deficits were found to be predominantly related to ADHD. Similar results have been obtained in studies using community samples [14, 53]. It is possible that EF deficits are associated primarily with attentional symptoms that exacerbate defiant behavior problems.

The opposite associations for CBCL internalizing symptoms and externalizing and attention symptoms probably led to EFs not being associated with preschool total psychiatric symptoms, which highlights the importance of studying different types of psychiatric symptoms separately.

In this study preschool age EFs did not predict school-age psychiatric symptoms, apart from CBCL attention symptoms. This is at odds with prior research [8, 10, 25]. A likely reason for the lack of longitudinal associations between EFs and psychiatric symptoms is our sample being clinical. Besides ADHD studies, most of the existing studies on preschool aged children and EFs have used community samples. In a community sample, children with deficiencies in EFs may be more likely in time to develop psychiatric symptoms, compared to children with optimal EFs. However, in a clinical sample, the symptoms are already present, and therefore the differences in EFs may not be as relevant predictor for future symptoms. In addition, the children in our sample were receiving psychiatric treatment already in preschool, which may have led to decrease of existing symptoms by the time they were in school—and thus be an indicator of the importance of early interventions. Another explanatory factor for decrease in symptoms could also be the increase in children’s ADHD medication from T1 (2.9%) to T2 (40%). Perhaps following the development of EFs across time would reveal more differences in the symptoms of child psychiatric patients, as the development of EFs has been suggested to be more relevant for later behavior problems than the actual level of EFs [54].

As hypothesized, deficits in preschool age total EF, inhibition, attention, and execution of action were all associated with concurrent ADHD diagnosis. This is in line with previous research [7, 8, 17, 21, 49]. Preschool age EFs—total, inhibition, attention, and execution of action—also predicted school age ADHD diagnosis. Even after controlling for preschool age diagnosis, total EF and execution of action remained significant predictors. Previous research on the matter has been conflicting, with some studies finding a longitudinal relationship [28–30] and others not [31, 32]. It should be noted that all of these earlier studies have used laboratory tasks (e.g. Go/No-go, Stroop, Tower of Hanoi) to measure EFs. Laboratory tasks appear to have low sensitivity to capture ADHD symptoms [19, 55] and they often yield diverging results from behavioral assessment (e.g., BRIEF, ATTEX) [19, 56]. Besides adding to the evidence on the role of EF deficits in ADHD, this study emphasizes the ability of daycare teachers to recognize these early EF deficits. It shows that assessing EFs with an ecologically valid behavioral measure filled in by daycare teachers can help in the early recognition of EF deficits related to later ADHD symptoms and thus enable early intervention.

In the existing longitudinal studies ADHD has typically been predicted by total EF, not the individual components [30–32, 34]. The current study offers an interesting look at the developmental continuity of EFs and ADHD. Although the differences were relatively small, it should be noted that of the individual EF components, execution of action had the strongest association with school age ADHD, whereas inhibition and attention did not significantly predict later ADHD after controlling for the preschool age diagnosis. With preschool ADHD, inhibition had the strongest association and execution of action the weakest. It might be that the skills of planning, initiation and execution of action are not as much required from preschool age children as those of inhibition and impulse control. Therefore, children receiving an ADHD diagnosis already in preschool probably have predominantly impulsive symptoms, whereas children receiving the diagnosis later might have difficulties in the higher EFs. Whether this reflects the manifestation of the different types of ADHD (inattentive, impulsive/hyperactive) is a question for future research.

## Limitations

This study has some limitations that need to be considered. First, the response rate of the follow-up phase remained moderate, 40.4% of the baseline participants answered after a total of three reminders. Although the samples did not differ from each other at T1 in the measures used in this study, there is still a possibility that the situation had changed for better or worse in the few years between the timepoints. Also, as the follow-up sample size remained only moderate, some differences and associations may not have been observed, as they would lack statistical power. Another limitation is the lack of data on EFs at the follow-up. Although ATTEX questionnaire was attempted to be collected from the teachers, the response rate remained so low that the data could not be used. In future longitudinal studies it would be informative to have comparable data of children’s EFs at the follow-up to be able to fully understand the associations. It has been reported that the development of EFs across time is more relevant for later behavior problems than the actual level of EFs [54].

In addition to the sample size, the sample characteristics also set some limitations. Although the child psychiatric sample is a definite strength, as there is a shortage of similar studies, it also limits the generalization of the results. The large proportion of boys and children with ADHD diagnosis in the sample also limits the generalizability of the results to female child psychiatric patients or those with mainly internalizing symptoms. Although we controlled for the effect of age, the relatively broad age range of participants (4–7 years at T1, 8–13 years at T2) may also have affected the results. Since the development of EFs is particularly rapid in preschool age [1], it can be assumed that an age difference of three years leads to very different EF profiles and deficits. In fact, [28] found that even between ages five and six there was a significant difference in the ability of simple inhibition to predict later ADHD symptoms. As the current study found several significant associations between preschool EFs and psychiatric symptoms and ADHD, it would be expected that the relationships would be even more pronounced in stricter age groups.

Finally, the methods used set some limitations on the interpretation of the results. Longitudinal associations do not equal causal relationships. It is possible that preschool age EFs do not actually precede school age attention symptoms or ADHD, but share common etiology instead (see [12]. Measuring EFs with a behavioral measure (ATTEX-P) has its limitations as well. While yielding ecologically valid data, observations are always subjective, and they lack the precision of laboratory tasks. At T2, ADHD diagnosis group included children with a verified ADHD diagnosis and no ADHD group children with other diagnoses and children of whom the T2 diagnoses were unavailable. This may bias the found associations between EFs and ADHD at T2.

## Conclusion

This study examined both concurrent and longitudinal relations of EFs with psychiatric symptoms and ADHD in preschool aged child psychiatric patients. It is one of the few studies investigating these associations in an actual clinical sample, and to our knowledge the first to look at the broad range of psychiatric symptoms in a patient group, not just in a specific diagnostic group.

It was found that although there were significant associations between preschool EFs and concurrent internalizing, externalizing and attention symptoms, the preschool EFs did not predict school age psychiatric symptoms, apart from attentional symptoms. Interestingly, the effects of inhibition and total EFs on concurrent internalizing and externalizing symptoms were opposite.

The results of the current study emphasize the role of EF deficits in ADHD, as they not only were associated with concurrent ADHD diagnosis, but also predicted school age ADHD, even after controlling for earlier diagnosis. In addition, the separate and slightly differing effects of EF components (inhibition, attention, execution of action) on ADHD diagnosis at the two timepoints suggest that different EF deficits may be relevant at different ages.

Future research should look more into the development of EFs across time and how it affects the manifestation of psychiatric symptoms in child psychiatric patients. The role of different EF components in the recognition of ADHD at different ages should also be further investigated. Based on the results of this study, a behavioral measure filled in by daycare teachers may offer valuable information in recognizing EF deficits related to ADHD symptomology already in preschool aged children. With more comprehensive understanding of a young child’s symptoms, more accurate support can be arranged.

## Summary

Child psychiatric patients were followed up to find out whether preschool age executive functions (EFs) associate with concurrent and school age psychiatric symptoms and ADHD diagnosis. EFs were measured at baseline with Attention and Executive Function Rating Inventory – Preschool Version, psychiatric symptoms were measured at both timepoints by Child Behavior Checklist. Information on diagnoses was collected from medical records. Preschool age deficits in EFs were associated with more concurrent externalizing and attention symptoms, and less internalizing symptoms, but predicted only attention symptoms at school age. Preschool EFs were associated with both concurrent and school age ADHD diagnosis. Our results emphasize the importance of recognizing EF deficits early to arrange appropriate support to reduce later problems.

## Data Availability

Data includes information on hospital patients and based on ethical approval and current laws cannot be accessed by other researchers than the authors who have been granted the hospital research permissions.
